# Brief Treatment of Symptoms of Post-Traumatic Stress Disorder (PTSD) by Use of Accelerated Resolution Therapy (ART^®^)

**DOI:** 10.3390/bs2020115

**Published:** 2012-06-18

**Authors:** Kevin E. Kip, Carrie A. Elk, Kelly L. Sullivan, Rajendra Kadel, Cecile A. Lengacher, Christopher J. Long, Laney Rosenzweig, Amy Shuman, Diego F. Hernandez, Jennifer D. Street, Sue Ann Girling, David M. Diamond

**Affiliations:** 1 College of Nursing, University of South Florida, Tampa, FL 33612, USA; E-Mails: celk@health.usf.edu (C.A.E.); kbarber@health.usf.edu (K.L.S.); rkadel@health.usf.edu (R.K.); clengach@health.usf.edu (C.A.L.); clonglcsw@gmail.com (C.J.L.); yenal3523@yahoo.com (L.R.); sgirling@health.usf.edu (S.A.G.); 2 Western New England University, Springfield, MA 01119, USA; E-Mail: amyshuman17@yahoo.com; 3 Balanced Living Psychology, Tampa, FL 33626, USA; E-Mail: docdiego@earthlink.net; 4 Life Renewal Counseling, Tampa, FL 33544, USA; E-Mail: jstreet@liferenewalcounseling.org; 5 Research and Development Service, VA Hospital, Tampa, FL 33612, USA; E-Mail: ddiamond@usf.edu; 6 Departments of Psychology and Molecular Pharmacology and Physiology, Center for Preclinical and Clinical Research on PTSD, University of South Florida, Tampa, FL 33612, USA

**Keywords:** psychological trauma, PTSD, exposure therapy, eye movements, bilateral stimulation, brief treatment

## Abstract

Post-Traumatic Stress Disorder (PTSD) is a prevalent, disabling anxiety disorder. This prospective cohort study reports on a new exposure-based therapy known as Accelerated Resolution Therapy (ART^®^) that incorporates the use of eye movements administered in a brief treatment period (1–5 one-hour sessions within three weeks). Eighty adults aged 21–60 years with symptoms of PTSD were recruited from the Tampa Bay area. The ART-based psychotherapy was designed to minimize anxiety and body sensations associated with recall of traumatic memories and to replace distressing images with favorable ones. Participants’ mean age was 40 years, 77% were female, and 29% were Hispanic. Participants underwent a median of three ART sessions, 66 of 80 (82.5%) completed treatment, and 54 of 66 (81.8%) provided 2-month follow-up data. Mean scores pre- and post-ART and at 2-month follow-up were: PTSD Checklist: 54.5 ± 12.2 *vs.* 31.2 ± 11.4 *vs.* 30.0 ± 12.4; Brief Symptom Inventory: 30.8 ± 14.6 *vs.* 10.1 ± 10.8 *vs.* 10.1 ± 12.1; Center for Epidemiologic Studies Depression Scale: 29.5 ± 10.9 *vs.* 11.8 ± 11.1 *vs.* 13.5 ± 12.1; Trauma Related Growth Inventory-Distress scale: 18.9 ± 4.1 *vs.* 7.4 ± 5.9 *vs.* 8.2 ± 5.9 (*p* < 0.0001 for all pre-ART *vs.* post-ART and 2-month comparisons). No serious adverse events were reported. ART appears to be a brief, safe, and effective treatment for symptoms of PTSD.

## 1. Introduction

Post-Traumatic Stress Disorder (PTSD) is a prevalent, disabling anxiety disorder that may occur after witnessing a traumatic event which then evokes a combination of re-experiencing, avoidance, numbing, and/or arousal symptoms [1]. Among adults 18 and older in the U.S. population, lifetime and past year prevalence of PTSD have been estimated at 6.8% and 3.5%, respectively [2]. The prevalence of PTSD varies considerably among subgroups, and is much higher than the national average among women [3], U.S. service members [4], and veterans [5,6]. Comorbidity rates of PTSD with other medical, mental health, and substance abuse disorders may exceed 80% [2]. The most common disorders seen in PTSD patients are depressive disorders, panic disorder, other anxiety disorders, substance abuse or dependence, and personality disorders [6,7,8]. In addition, PTSD is associated with indicators of poor general health—increased physical symptoms, high somatic symptom severity, and higher numbers of sick call visits and missed workdays [9]. This high prevalence of psychological and physical comorbidities is associated, in turn, with impaired family relationships, work relationships, and friendships [10], which may contribute to the reported increased risk of suicidal behavior [11]. 

First line evidence-based cognitive-behavioral treatments (CBT) for PTSD include Cognitive Processing Therapy (CPT), Prolonged Exposure (PE) therapy, and Eye Movement Desensitization and Reprocessing (EMDR) which lead to clinically improved outcomes in approximately 50% of all treated cases [12,13,14]. CPT, which entails 12 treatment sessions, focuses on challenging and modifying maladaptive beliefs related to the trauma, while including a written exposure component [15]. PE, which includes imaginal and *in vivo* exposure to safe situations that have been avoided because they elicit traumatic memories, typically requires 8–15 treatment sessions [16]. EMDR [17,18] engages in imaginal exposure to a trauma while simultaneously performing saccadic eye movements. It is an 8-phase program that entails 5–15 treatment sessions. In addition to CBT, pharmacological treatment with selective serotonin reuptake inhibitors (SSRI’s) has been specifically approved by the FDA for treatment of PTSD [19]. However, SSRI’s usually do not eliminate all symptoms, and potential side effects need to be continuously monitored during use [20]. 

Overall, no single approach has been shown to be completely efficacious in the treatment of broad range of symptoms in PTSD. Hence, there remains intense interest in exploring new ways to advance PTSD treatment effectiveness, accessibility, and delivery. This includes drawing on strengths of individual therapies, and ideally, reducing overall treatment duration. These conditions led to the development of a new form of exposure-based therapy in 2008 known as Accelerated Resolution Therapy (ART^®^) that uses eye movements and is the focus of this report. This therapy was developed to be highly procedural (standardized) and to be administered to patients in a shorter period of time than first-line evidence-based treatments for PTSD (1–5 sessions in 3 weeks). 

## 2. Materials and Methods

### 2.1. Participant Inclusion and Exclusion

Beginning in May of 2011, a federally-funded prospective cohort treatment study of adults with symptoms of PTSD was initiated at the University of South Florida (USF) in Tampa, FL. Participant inclusion criteria were: (i) between the age of 21–60 years inclusive; (ii) symptoms indicative of PTSD, as defined as a score of >40 on the PTSD Checklist (range 17–85)—civilian version (PCL-C) [21,22], or in the absence of a score >40, other documented evidence of symptoms, including a high PTSD subscale score and/or endorsement of specific PTSD item responses on the Psychiatric Diagnostic Screening Questionnaire (PDSQ); (iii) ability to read and speak English to complete survey questions; and (iv) denial of suicidal and homicidal ideation or intent and no evidence of psychotic behavior or otherwise being in psychological crisis. Exclusion criteria were: (i) brain injury prohibiting speech, writing, and purposeful actions; (ii) current suicidal ideation; (iii) major psychiatric disorder primary to symptoms of psychological trauma and anticipated to interfere with treatment (e.g., schizophrenia, bipolar disorder); (iv) current treatment for substance abuse; (v) previous diagnosis of eye movement disorder anticipated to interfere with treatment (e.g., amblyopia); and (vi) any medical condition that, in the judgment of the Principal Investigator and/or ART therapist, may place the individual at high risk due to a potential heightened emotional reaction (e.g., previous heart attack, seizure disorder). Individuals with previous treatment for symptoms of PTSD, yet with residual symptoms that met inclusion criteria upon screening, were eligible for the study.

### 2.2. Recruitment

Participant recruitment occurred through caseload referrals from licensed mental health therapists in the study area, flyers and brochures, direct meetings with providers of mental health services, and as a result of local media coverage of ART. All participants were treated at the USF College of Nursing and received $20 for completion of self-report questionnaires (see below) before and after treatment and at 2-month follow-up. The study protocol was approved by the USF Institutional Review Board and all participants provided written informed consent.

### 2.3. Therapist Training

All therapists underwent intensive training in ART conducted in person by the developer (L.R.) and lead trainer (A.S.) and in accordance with the ART training manual. This included two 8-hour days on the theory, principles, and protocol for conducting ART including intake assessment, intervention techniques, eye movement regimen, challenges and solutions, and closure techniques. This was followed by directly-observed supervised practice, and then follow-up training and assessment. A total of 8 therapists conducted the ART sessions including: 1 Clinical Psychologist (Psy.D.); 4 licensed mental health counselors (LMHC); 2 licensed clinical social workers (LCSW); and 1 licensed marriage and family therapist (LMFT). Therapists included males and females and those of Hispanic and non-Hispanic ethnicity.

### 2.4. Participant Intake Assessment

Participants completed 3 screening instruments as part of their intake assessment. This included the 17-item PCL-C Checklist [21,22], a self-developed 9-item ART Intake Questionnaire, and the 125-item (yes/no) PDSQ. The PDSQ was used to screen for Axis I disorders and serve as a baseline assessment of psychopathology. This instrument has been validated against diagnostic criteria and interview-derived diagnoses over the course of 10 years and more than 3,000 administrations [23,24]. It can be quickly hand scored to obtain a total score (which functions as a global indicator of psychopathology) plus scale scores for 13 disorders: Major Depressive Disorder, Generalized Anxiety Disorder, Panic Disorder, PTSD, Alcohol Abuse/Dependence, Drug Abuse/Dependence, Psychosis, Bulimia/Binge-Eating Disorder, Somatization Disorder, Obsessive-Compulsive Disorder, Social Phobia, Hypochondriasis, and Agoraphobia. Six items on the depression scale provide a measure of suicidal ideation. In general, a T-score of 40–60 is consistent with the level of symptomatology typically seen in the outpatient population, and indicates the presence of significant psychological distress (*i.e.*, need for treatment). “Screen fail” participants (*i.e.*, those not meeting enrollment criteria, including those with a T-score >70 indicating need for a higher level of care) were offered a direct referral for counseling in the community, or 2 complimentary sessions of an empirically-based method of psychotherapy at the study site (and appropriate referral thereafter).

### 2.5. ART Protocol

The ART protocol is theoretically grounded in and uses cognitive behavioral and experiential therapies along with psychodynamic psychotherapy. ART was developed to treat both physiological and cognitive aspects of PTSD, which as a disorder, has been described as a consequence of failed memory processing when the brain fails to appropriately consolidate and integrate *episodic* memory into the *semantic* memory system [25]. This breakdown of the normal process of memory transfer and integration has been proposed to lead to continued maintenance of the episodic memory and its affect in an inappropriately strong and affect-laden form [25]. By protocol, the repeated use of sets of eye movements in ART are conducted to facilitate the separation (elimination) of physiological sensations associated with purposeful recall of traumatic experiences prior to cognitive intervention. Then, a technique known as Voluntary Image Replacement (VIR) is used “replace” the distressing images (but not narrative memory) with more pleasing images. Thus, it is postulated that ART aims to transfer and integrate episodic memories from the hippocampus into the neocortex, an activity that may efficiently occur after the heightened physiological responses invoked by exposure therapy have been minimized (*i.e.*, the subject is no longer in “fight or flight” mode, and neocortical integration can occur). The use of VIR parallels Imaging Rescripting (IR) (“Type A”) in which a preexisting negative mental image is transformed into a more benign image (*i.e.*, negative image to positive image through rescripting) [26], and which has been successfully used to treat survivors of traumatic industrial accidents suffering from PTSD [27].

Thus, unique features of ART include: (i) a central focus on reconsolidating disturbing memories; (ii) a fixed number of eye movements (sets of 40) to reduce physiological and affective responses during focused recall of events; (iii) continuous assessment of physiological sensations and images targeted for cognitive reduction/removal; and (iv) patient directed re-envisioning of events in a resolving narrative with the VIR. Despite similarities with EMDR, the most poignant differences between ART and EMDR are: (i) *Images*: ART uses the VIR technique to change the actual recall of images (*i.e.*, from negative to positive), whereas EMDR aims to cognitively desensitize the client about their trauma (images); (ii) *Sensation processing*: ART spends considerably more time processing physiological sensations than EMDR, and by protocol, dictates that after each “scene-focused” set of eye movements, the therapist use a corresponding set of eye movements specifically to process (remove) physiological sensations; (iii) *Standardization*: For each set of eye movements, ART uses a fixed number (40) to help the client process, but not be flooded with information, whereas EMDR changes the number of eye movements. In addition, ART clinicians use a set of standard interventions (e.g., the “Director”) and a fidelity checklist from the ART training manual, whereas EMDR is less standardized and may require the therapist to come up with their own “Cognitive Interweave” when they get “stuck”. Thus, unlike EMDR, ART is purposely not free-associative. 

The length of treatment with ART is based on processing of one or more traumatic scenes identified as contributing to symptoms of PTSD. An average of three representative scenes may be processed to eliminate presenting sensations and symptoms, with an apparent generalizing effect for any remaining scenes. Thus, treatment is completed when the scene or representative scenes are processed and the participant reports significant or full relief from these scenes. Depending on circumstances, it is possible to process up to three scenes in a one hour session.

### 2.6. Individual ART Sessions

Participants underwent between 1–5 sessions of ART (within 3 weeks), each approximately 60 to 75 minutes in length. In brief, the 2 major components of each ART session were: (i) minimize or eliminate physiological response associated with recall of the traumatic memory(ies); and (ii) re-envisioning of events (images) with the VIR technique. Thus, individual sessions included: (i) initial assessment of Subjective Units of Distress (SUDS) on a 10-point scale; (ii) discussion with the participant on the intended use of the VIR for the distressing scene(s) to be treated; (iii) with use of lateral eye movements, reduction or elimination of anxiety and/or somatic sensations associated with purposeful recall of the traumatic memory(ies); (iv) therapist-directed implementation of a creative intervention(s) (from the ART manual) to achieve the VIR; (v) attempted recall of the original distressing scene *versus* the new scene to assess participant response to the VIR; (vi) therapist-directed discussion about any future events anticipated to trigger the symptoms associated with the original memory and, if found, implementation of an ART intervention to neutralize their potential potency; (vii) closure assessment, to include discussion of any future traumatic memories to be treated in subsequent ART sessions; and (viii) session closeout assessment of SUDS on 10-point scale. The total number of ART sessions required for treatment completion varied by participant, and was based on the number and length of traumatic scenes to be processed (*i.e.*, those associated with PTSD), including “new” scenes identified during processing of the index scene(s) for which the participant originally sought treatment. 

### 2.7. Data Collection

After written consent and clinical screening for determination of study eligibility, participants completed a demographic and brief medical history questionnaire. Baseline completion of self-reported outcome measures (in addition to the previously completed PCL-C) included the following measures: 18-item Brief Symptom Inventory (BSI) [28]; 20-item Center for Epidemiologic Studies Depression Scale (*CES**-**D) *[29]; 21-item State-Trait Inventory for Cognitive and Somatic Anxiety (STICSA) [30]; 26-item Self-Compassion Scale (SCS) [31]; 29-item Aggression Questionnaire (AQ) [32]; 10-item Alcohol Use Disorder Identification Test (AUDIT) [33]; 32-item Trauma-Related Guilt Inventory (TRGI) [34]; 21-item Post-Traumatic Growth Inventory (PTGI) [35]; and the Pittsburgh Sleep Quality Index (PSQI) [36]. These measures were selected to assess a wide range of psychological treatment response and on the basis of established reliability and validity. The outcome measures were completed immediately before the first ART session, after the final ART session, and at 2 months post-treatment. Occurrence of adverse events was inquired by the treating therapist prior to each ART session including the nature and intensity of each event, subsequent treatment actions, and judgment as to whether the event was causally related to use of ART.

### 2.8. Statistical Methods

Continuous variables are expressed as mean ± standard deviation (SD); categorical variables are presented as percentages. Paired *t* tests were used to compare changes in the above defined outcome measures before and after treatment completion and at 2-month follow-up. Standardized effect sizes for outcome measures were calculated as: ((mean before ART − mean after ART)/standard deviation of treatment difference scores) [37]. McNemar’s test for paired data was used to compare the proportion of subjects using medications, as well as those above established clinical cutoff scores for PTSD, depression, and sleep dysfunction before and after treatment with ART and at 2-month follow-up. In sensitivity analyses, analysis of covariance (ANCOVA) was used to compare initial treatment response between participants with and without 2-month follow-up data. Due to multiple comparisons, a “corrected” two-tailed *p* value of <0.01 was used to define statistical significance. 

## 3. Results

### 3.1. Screening and Enrollment

**Figure 1:**
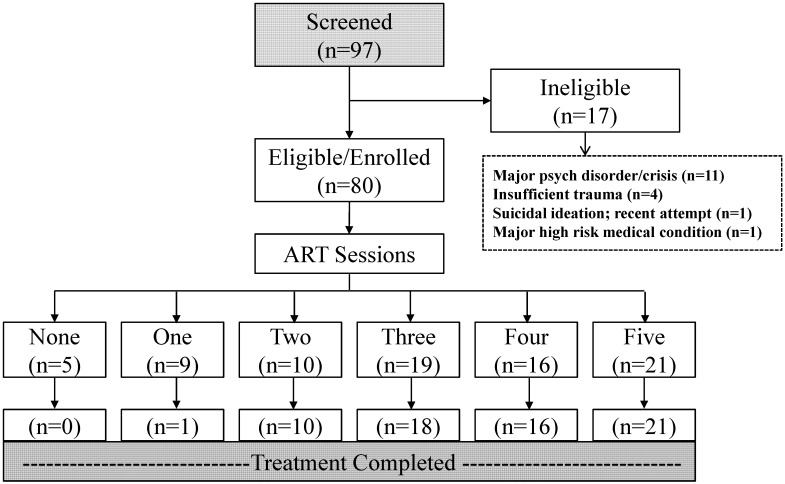
Flow diagram of screening, enrollment, and treatment completion of study participants.

A total of 97 persons were screened, of whom, 17 (17.5%) were determined to be ineligible for the study. Primary reasons for ineligibility included major concomitant psychiatric disorder primary to psychological trauma or being in psychological crisis (n = 11), and subclinical levels of symptomatology of psychological trauma (n = 4) (Figure 1). Among the 80 enrolled participants, 66 (82.5%) completed the full course of treatment including initial post-treatment assessment. The majority (64%) of participants who did not complete treatment stated work and personal schedule conflicts. The median number of ART sessions held was three for participants enrolled and four for those enrolled and who completed treatment. 

Principal reasons identified for non-completion were: (i) logistical and scheduling conflicts (n = 3); (ii) advice/interaction by another mental health professional (n = 2); difficulty in performing eye movements (n = 1, participant had a history of surgically-corrected amblyopia); and other/unspecified reasons (n = 8). None of the 14 participants who did not complete treatment specified the occurrence of adverse effects. Among the 66 participants who completed the full course of treatment, 54 completed 2-month follow-up assessments and 12 were lost to follow-up. Most participants lost to follow-up stated work and personal schedule conflicts, as well as limited financial incentive from taking the time to complete questionnaires.

### 3.2. Demographic and Presenting Characteristics

The mean age of study participants was 40.0 ± 10.2 years, 77% were female, 89% were of Caucasian race, and 29% were of Hispanic ethnicity (Table 1). The mean PDSQ T-score at study entry was 53.7 ± 8.6 indicating, on average, clinically significant psychopathology, as well as high PTSD symptomatology as evidenced by a mean PCL-C score of 54.5 ± 13.0. Eleven participants (13.7%) had PCL-C scores below 40 yet were enrolled in the study based on their PTSD subscale score on the PDSQ meeting the cutoff score of ≥5 (10 of 11 participants), and all 11 participants endorsing at least 1 of the “critical” DSM-IV PTSD items. Of note, approximately half of all participants reported ≥5 traumatic memories still impacting their life, 80% reported living with traumatic memories for ≥7 years. The trauma(s) for which treatment was sought was classified as experiencing violent or abusive crime (51%), loss of a loved one (29%), divorce (11%), chronic or acute illness (10%), and other. Medication use at study entry was common and highly variable. This included current use of anti-anxiety medications (32.5% of all participants), anti-depressants (27.5%), pain medications (8.7%), sleep medications (7.5%), and anti-seizure medications (7.5%). 

**Table 1:** Demographics and Presenting Characteristics.

Characteristic		All (n = 80)	Male (n = 18)	Female (n = 62)
Age in years (mean ± SD)		40.0 ± 10.2	41.9 ± 11.8	40.6 ± 9.8
Race (%)				
	Black or African American	7.5	0.0	9.7
	White	88.8	94.4	87.1
	Asian	1.3	0.0	1.6
	Native Hawaiian/Pacific Islander	0.0	0.0	0.0
	American Indian/Alaskan Native	1.3	0.0	1.6
	Not reported	1.2	5.6	0.0
	Hispanic (%)	28.6	5.6	35.6
	Married (%)	58.8	61.1	58.1
Employed – full or part time (%)		59.8	72.2	55.9
PDSQ score (mean ± SD) (T-score)		53.7 ± 8.6	54.6 ± 8.6	53.5 ± 8.6
	Less than 40 (%)	2.5	0.0	3.2
	40 to 60 (%)	75.0	77.8	74.2
	More than 60 (%)	22.5	22.2	22.6
PCL-C score (mean ± SD)		54.5 ± 13.0	57.3 ± 14.3	53.7 ± 12.6
	Less than 40 (%)	13.8	11.1	14.5
	40 to 60 (%)	47.5	38.9	50.0
	More than 60 (%)	38.8	50.0	35.5
No. of traumatic memories still impacting life (%)				
	1 to 2	19.0	23.57	17.7
	3 to 4	31.6	35.3	30.6
	5 or more	49.4	41.2	51.6
Previous treatment for PTSD/other MH condition (%)		67.9	50.0	73.3
On disability for PTSD or other MH disorder (%)		10.1	5.9	11.3
Guilt associated with traumatic memory(ies) (%)		81.3	88.9	79.0
Time lived with traumatic memory(ies) (%)				
	Less than 1 year	6.3	5.6	6.5
	1 to 6 years	13.8	22.2	11.3
	7 years or more	80.0	72.2	82.3

PDSQ: Psychiatric Diagnostic Screening Questionnaire; PCL-C: PTSD Checklist, Civilian Version; MH: Mental Health.

### 3.3. Examination of Treatment Efficacy

For the 54 subjects who completed treatment and had 2-month follow-up data, very large, statistically significant initial treatment effects were observed across a range of symptom measures (Table 2, left side). For the primary outcome of PTSD symptoms, the mean score on the PCL-C was 54.5 ± 12.2 before ART *versus* 31.2 ± 11.4 after ART (mean difference = 22.8 ± 13.5; effect size = 1.72; *p* < 0.0001). In rank order, the largest effect sizes observed were for the following measures: TRGI-Distress subscale (effect size = 1.88), BSI (effect size = 1.74), PCL-C (effect size = 1.72), STICSA-Cognitive Anxiety subscale (effect size = 1.62), and CES-D (effect size = 1.41) (*p* < 0.0001 for all measures). Large clinical improvements in symptoms were also reported for subscales of the PTGI, STICSA-Somatic Anxiety subscale, PSQI, TRGI global guilt and guilt cognition subscales, and SCS (*p* < 0.0001 for all measures).

In examining sustainability of treatment effects, symptom measure scores at 2 month follow-up were essentially parallel to those reported after treatment completion (Table 2, right side). In rank order, the largest pre-treatment to 2-month follow-up effect sizes observed were for the following measures: PCL-C (effect size = 1.98), TRGI-Distress subscale (effect size = 1.88), BSI (effect size = 1.57), and CES-D (effect size = 1.46) (*p *< 0.0001 for all measures). Importantly, symptom scores did not differ statistically (at *p* < 0.01) between any of the initial post-treatment *versus* 2-month follow-up assessments with the exception of the STICSA-Cognitive Anxiety subscale score being nominally higher at 2 months (15.5 ± 5.0 *versus* 17.5 ± 6.8; *p* = 0.01), and conversely, alcohol use as measured by the AUDIT being nominally lower at 2 months (2.5 ± 3.0 *versus* 1.8 ± 2.1; *p* = 0.0008). Thus, results obtained post-treatment and at 2-month follow-up can be interpreted as approximately equivalent.

### 3.4. Subgroup Analyses

**Figure 2:**
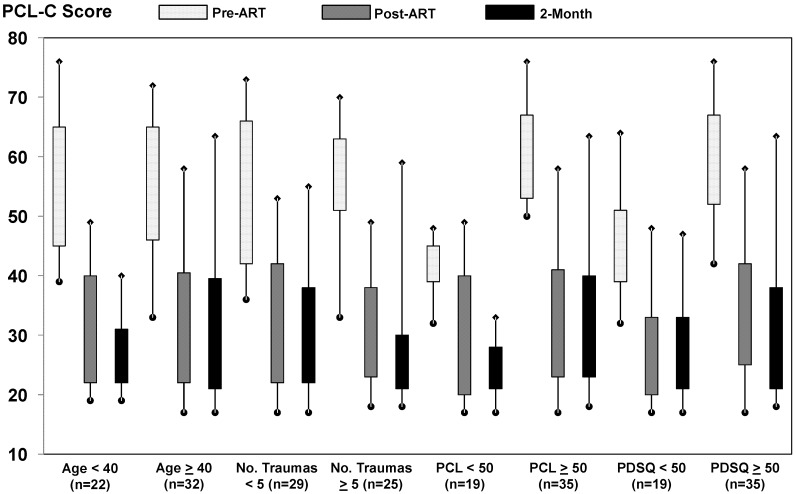
Distribution of self-report scores on the PCL-C among subgroups at baseline, post-treatment, and 2-month follow-up. The rectangles depict the interquartile range; the lower and upper ends of the vertical lines with the diamonds depict the 5th and 95th percentiles, respectively.

**Figure 3:**
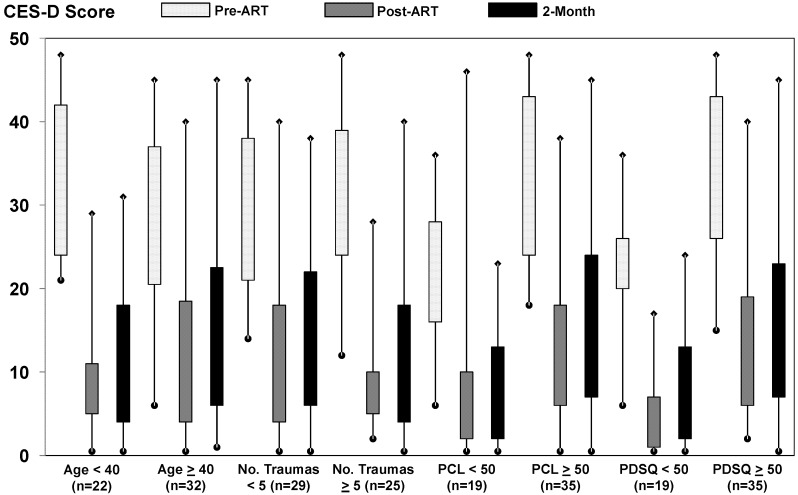
Distribution of self-report scores on the CES-D among subgroups at baseline, post-treatment, and 2-month follow-up. The rectangles depict the interquartile range; the lower and upper ends of the vertical lines with the diamonds depict the 5th and 95th percentiles, respectively.

In subgroup analyses by age (<40 *vs.* ≥40 years), number of reported traumas still impacting life (<5 *vs.* ≥5), presenting PCL-C score (<50 *vs.* ≥50), and presenting PDSQ-T score (<50 *vs.* ≥50), large, clinically significant reductions in symptoms of PTSD were consistently reported after treatment completion and at 2-month follow-up (Figure 2* p* < 0.001 for all comparisons with pre-treatment scores). For participants who presented with a PDSQ-T score of <50 (n = 19), respective before ART, after ART, and 2-month mean follow-up scores on the PCL-C were 44.6, 26.6, and 27.1. For participants who presented with a PDSQ-T score of >50 (n = 35), respective before ART, after ART, and 2-month mean follow-up scores on the PCL-C were 59.9, 33.7, and 31.6. Similar consistent and sustained treatment effects were observed for reductions in symptoms of depression across all subgroups examined (Figure 3* p* < 0.001 for all comparisons with pre-treatment scores). Although there was some overlap in the 95% confidence intervals for pre- and post-treatment PTSD and depression scores, this appeared to be due to imprecision in subgroup analyses (*i.e.*, small sample sizes), as effect sizes were consistent with those observed among the full cohort. In addition, favorable treatment effects were observed across all of the 8 treating therapists and the number of treatment sessions provided (data not shown).

**Table 2:** Self-Report Changes in Symptoms Before and After Treatment with ART.

Symptom Measure	Pre- *Versus* Post-Treatment						Pre-Treatment *Versus* 2-Month Follow-Up					
	N	Pre ^a^	Post ^a^	Diff ^ab^	ES	P	N	Pre ^a^	2-Mo. ^a^	Diff ^ab^	ES	P
PTSD Checklist (PCL-C)	54	54.5 (12.2)	31.2 (11.4)	22.8 (13.5)	1.72	<0.0001	54	54.5 (12.2)	30.0 (12.4)	24.5 (12.4)	1.98	<0.0001
Brief Symptom Inventory	52	30.8 (14.6)	10.1 (10.8)	20.8 (11.9)	1.74	<0.0001	54	30.7 (14.3)	10.1 (12.1)	20.7 (13.1)	1.57	<0.0001
CES-D (Depression)	54	29.5 (10.9)	11.8 (11.1)	17.7 (12.5)	1.41	<0.0001	54	29.5 (10.9)	13.5 (12.1)	16.0 (11.0)	1.46	<0.0001
STICSA (Somatic)	54	20.6 (6.9)	13.8 (3.5)	6.9 (6.2)	1.11	<0.0001	54	20.6 (6.9)	14.9 (5.0)	5.7 (5.2)	1.10	<0.0001
STICSA (Cognitive)	54	25.2 (6.6)	15.5 (5.0)	9.7 (6.0)	1.62	<0.0001	54	25.2 (6.6)	17.5 (6.8)	7.7 (6.8)	1.14	<0.0001
Pittsburgh Sleep Quality	46	9.2 (4.5)	6.4 (4.4)	2.4 (3.1)	0.87	<0.0001	45	9.3 (4.6)	7.0 (4.6)	2.4 (3.7)	0.70	<0.0001
Trauma Related Growth												
Global Guilt	54	4.7 (2.4)	1.6 (1.9)	3.1 (2.4)	1.28	<0.0001	54	4.7 (2.4)	1.9 (2.0)	2.8 (2.8)	1.02	<0.0001
Distress	54	18.9 (4.1)	7.4 (5.9)	11.2 (6.0)	1.88	<0.0001	54	18.9 (4.1)	8.2 (5.9)	10.7 (5.7)	1.88	<0.0001
Guilt Cognition	54	44.7 (18.6)	24.1 (13.2)	20.1 (17.1)	1.17	<0.0001	54	44.7 (18.6)	24.5 (15.2)	19.7 (19.0)	1.04	<0.0001
Post-Traumatic Growth												
I:Relation to Others	52	12.3 (6.9)	17.0 (6.6)	4.8 (4.4)	0.89	<0.0001	52	12.3 (6.9)	15.3 (6.8)	3.0 (4.9)	0.66	<0.0001
II: New Possibilities	52	12.7 (6.5)	16.9 (6.6)	4.4 (6.0)	0.72	<0.0001	52	12.7 (6.5)	15.5 (7.2)	2.8 (5.5)	0.53	0.0009
III: Personal Strength	52	8.5 (5.4)	11.8 (6.2)	3.6 (5.5)	0.63	<0.0001	52	8.5 (5.4)	11.6 (5.6)	3.1 (4.6)	0.69	<0.0001
IV: Spiritual Change	52	5.3 (2.9)	7.2 (2.6)	2.0 (2.7)	0.73	<0.0001	52	5.3 (2.9)	6.4 (3.0)	1.1 (3.1)	0.36	0.008
V: Appreciation-Life	52	7.8 (4.0)	9.6 (4.2)	2.8 (4.3)	0.44	0.001	52	7.8 (4.0)	9.3 (3.9)	1.5 (4.0)	0.39	0.009
Self-Compassion Scale	54	66.8 (16.8)	84.4 (21.4)	16.4 (19.9)	0.82	<0.0001	54	66.8 (16.8)	81.7 (21.8)	14.9 (17.8)	0.84	<0.0001
Aggression Questionnaire	54	77.2 (20.2)	63.9 (20.1)	13.2 (13.5)	0.98	<0.0001	54	77.2 (20.2)	64.5 (21.0)	12.7 (15.0)	0.85	<0.0001
Alcohol Use (AUDIT)	52	3.0 (3.3)	2.5 (3.0)	0.6 (1.8)	0.26	0.03	54	2.9 (3.3)	1.8 (2.1)	1.1 (2.3)	0.48	0.0008

^a ^Presented as mean (standard deviation); ^b ^All mean differences are coded with positive numbers reflecting improvements in symptoms; ES: effect size. All comparisons of symptom measure scores at post-treatment *versus* 2-month follow-up were not statistically significant at P < 0.01 (specified level for statistical testing) with the following exceptions: STICSA-Cognitive (p = 0.01); Alcohol Use-AUDIT (P = 0.005).

### 3.5. Clinical Interpretation of Treatment Effects

**Figure 4:**
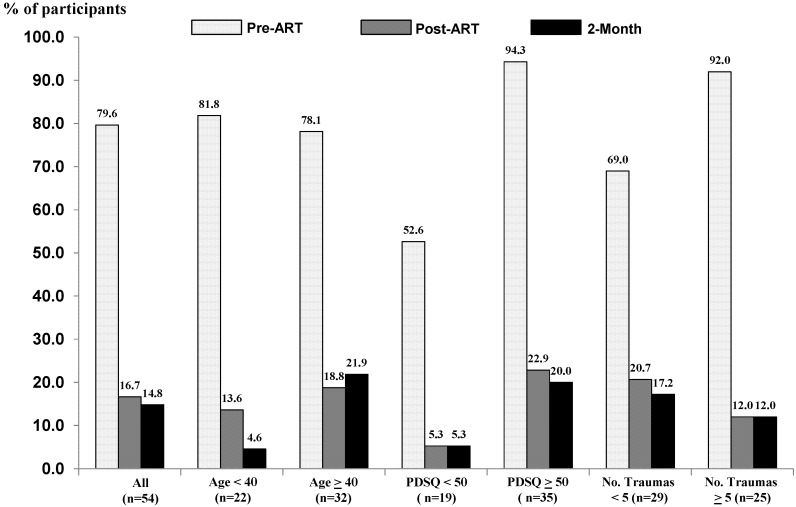
Percentage of participants with PCL-C scores >44 (established clinical cutpoint) at baseline (minimal shading), post-treatment (moderate shading) and 2-month follow-up (dark shading).

The instruments used in this study measured symptom burden, as opposed to deriving formal diagnoses. However, for the PCL-C, various cutoff scores exist for screening or diagnosis of PTSD among different settings. A score of ≥44 has been shown to maximize diagnostic efficiency [21]. Using this cutoff score, respective percentages of participants meeting this diagnostic criterion of PTSD before ART, after ART, and at 2-month follow-up were 80% *versus* 17% *versus* 15% (Figure 4, *p* < 0.001 for all comparisons with pre-treatment scores). Similar results were observed in subgroups classified by age, presenting PDSQ score, and number of reported traumas still impacting life. A second way to “diagnose” PTSD using the PCL-C is by specific symptoms rated as “Moderately” or above, and requiring at least 1 B item (questions 1–5), 3 C items (questions 6–12), and at least 2 D items (questions 13–17) [22]. Using this definition, 61 of 80 enrolled participants (76.3%) had PTSD, of whom, 50 (82%) completed treatment. Of these 50 participants, 76% and 74% no longer met the definition of PTSD after treatment and at 2-month follow-up, respectively. A third way to “diagnose” and assess PTSD treatment response is based on a score of ≥54, as determined by receiver operating characteristics (ROC) analysis in public sector mental health settings [38]. For the 26 participants with a PCL-C presenting score of ≥54, the mean reduction (difference) from baseline on the PCL-C was substantial after treatment completion (30.3 ± 12.0; effect size = 2.51, P < 0.0001) as well as at 2-month follow-up (29.7 ± 14.4; effect size = 2.06, P < 0.0001).

For the CES-D, scores of ≥16 are traditionally interpreted as suggestive of clinically significant depression [39]. Respective percentages of participants meeting this definition of depression before ART, after ART, and at 2-month follow-up were 90% *versus* 28% *versus* 33% (*p* < 0.001 for all comparisons with pre-treatment scores). Therefore, while not formally diagnostic, treatment with ART resulted in substantial reduction in symptoms with a majority of participants scoring below diagnostic cutoff scores for PTSD and depression after treatment and at 2-month follow-up. 

### 3.6. Medications

Small, non-significant reductions (P > 0.05) in the use of medications were reported comparing pre-treatment to 2-month follow-up. This included anti-anxiety medications (31.5% *vs.* 22.2%), anti-depressants (29.6% *vs.* 22.2%), medications for pain (7.4% *vs.* 5.6%), sleep medications (5.6% *vs.* 1.8%), and anti-seizure medications (9.3% *vs.* 7.4%). 

### 3.7. Adverse Events

No serious adverse events judged to be related to treatment with ART were reported during the course of treatment or during 2-month follow-up. During the treatment period (1–5 sessions), minor unrelated adverse events reported included headache and dizziness (n = 1), lightheadedness (n = 1), tension headache (n = 1), lack of motivation (n = 1), and waking during the night (n = 1). One participant reported mild depression after their first ART session which did not reoccur in subsequent sessions. A second participant reported feeling mildly depressed the day after an ART session.

### 3.8. Sensitivity Analyses

As described above, 12 of 66 participants (18.2%) who completed ART did not have 2-month follow-up data, thereby representing a potential source of bias for the 2-month results. In ANCOVA adjusted for baseline PCL-C score, adjusted post-treatment means were similar between the 54 participants with follow-up data *versus* the 12 participants who completed treatment but did not have follow-up data (31.0 *versus* 29.1, *p* = 0.57). Evidence of similar initial treatment response was observed for adjusted post-treatment mean scores on the CES-D (11.3 *versus* 13.4, *p* = 0.52) and BSI (9.7 *versus* 11.9, *p* = 0.44). In addition, 63 of 66 (95.5%) and 59 of 66 (89.4%) participants who completed treatment (irrespective of having follow-up data) had reductions of ≥5 and ≥10 points on the PCL-C from the baseline assessment, metrics that have defined to represent “reliable” and “clinically meaningful” change, respectively [40].Assuming the worst case scenario whereby all 14 participants who do not complete treatment failed to respond, this would result in rates of 78.7% and 73.7% for reliable and clinically meaningful change, respectively.

## 4. Discussion

### 4.1. Summary of Findings

In this first empirical report of ART, we observed substantial reductions in self-report symptoms of PTSD, depression, anxiety, and global physical and psychological symptoms, and improvements in trauma-related growth, sleep quality, and self-compassion after a median of 4 treatment sessions. Favorable results were consistently observed among all subgroups examined and at 2-month post-treatment follow-up. The majority of persons screened were judged to be eligible for the study, and approximately 83% of those enrolled were willing and able to complete the brief treatment regimen including completion of study questionnaires. In addition, no serious adverse effects were noted. Collectively, these results motivate future investigation of ART using controlled study designs, and possibly different clinical populations.

### 4.2. Possible Therapeutic Mechanism

Assuming that ART may be effective in resolving psychological trauma, the specific mechanism(s) by which this occurs is unknown. Within the realm of eye movement therapies, there is no consensus as to the therapeutic role of these movements. Existing hypotheses include those related to working memory [41], reciprocal inhibition [42], detached processing [43], inter-hemispheric integration [44], physiological effects [45], orienting response [46], and possible neurobiological mechanisms [18,25]. At the broadest level, we postulate that the protocol-specified sets of eye movements have a calming effect that may help to disengage the sympathetic response (e.g., increased heart rate, chest tightness, sweating, *etc.*) associated with recall of the traumatic memory and facilitate a reciprocal inhibition allowing enhanced information processing. This may occur in multiple ways.

First, the lateral left-right eye movements may simultaneously activate both hemispheres to foster inter-hemispheric communication in a “problem-solving” manner similar to that used during rapid eye movement (REM) sleep. Specifically, a hallmark of PTSD is disrupted sleep with intrusive, terrifying dreams and defective processing of emotionally laden memories [47]. The sets of eye movements with ART may “simulate” aspects of REM sleep. A proposed function of sleep-dependent memory consolidation is to discover the significance of a memory by facilitating its integration into a network of older, related memories [48], thereby providing a larger context and meaning for the event. Of note, both human [49] and animal [50] studies suggest that emotional memories are selectively processed during REM sleep, when more flexible and associative [51] cognitive processing occur. Images deleted (replaced) with ART may work in a similar manner as consolidation of information that takes place during REM sleep.

Second, as the patient seeks (is directed) to re-envision the traumatic event and direct a new narrative, the patient is able to imagine replacing images and sensations associated with the traumatic memory with self-selected images and sensations that are more pleasing and palatable. This process is strikingly similar to cognitive behavioral Imagery Rescripting techniques as described by Holmes, Arntz, and Smucker, where negative images are rescripted into positive images, or where an entirely new positive image may be created to address the patients’ concerns [26]. However, ART involves an additional therapeutic element known as the “Director” intervention that directs the patient to establish a new narrative to address “unfinished business” in much the way Gestalt techniques are used experientially to achieve positive results. Success of this intervention is determined by the therapist asking the participant to pull up the original distressful images, and reporting being unable to do so. 

### 4.3. Systemic Treatment Effects

While ART was delivered principally to resolve symptoms of PTSD, large concomitant reductions were observed for symptoms of depression and cognitive and somatic anxiety, along with marked improvements in trauma-related growth, sleep quality, and self-compassion. At least 3 possibilities exist for these widespread and somewhat “non-specific” effects. First, given that PTSD is associated with a range of psychological and physical comorbid conditions [7,9], “systemic” clinical benefits are to be expected so long as traumatic experiences of the patient are the principal underlying source of their psychopathology. On the other extreme, it is possible that, at least in part, “non-specific” effects were observed simply from subject interaction with therapists (e.g., the Hawthorne effect). The observation that the reported treatment effects were sustained at 2-month follow-up (which did not include interaction with a therapist) would argue against a broad Hawthorne effect. Third, many of the self-report measures used are correlated and perhaps favorably endorsed at large through a general sense of increased hope and reduced distress through interaction with a therapist. Whereas concomitant reported treatment improvements in self-compassion and lower aggression would appear as domains distinct from increased hope and reduced distress, the lack of a formal control condition precluded quantification of the net benefits achieved from ART.

### 4.4. Current Treatment Modalities

Guidelines from the recent Institute of Medicine (IOM) report on effective treatments for PTSD unanimously recommend cognitive behavioral therapies and most guidelines recommend EMDR [13,52]. While there is considerable empirical evidence of treatment effectiveness of PTSD with CBT (e.g., [53,54,55,56], and EMDR (e.g., [57,58]), these therapies (and other evidence-based modalities) may have significant limitations including: (i) lengthy treatment regimens (e.g., [59]) and (ii) significant post-treatment residual symptoms. The brevity of ART coupled with the very large, consistent, and sustained within-person effect sizes observed from this uncontrolled study provide a rationale for future controlled studies of this emerging therapy.

### 4.5. Strengths and Limitations

There are several strengths to our study. First, all therapists were formally certified in ART using a standard protocol, recruited from the community, and possessed varying educational backgrounds at the masters and doctoral level. This provides some degree of generalizability across therapists, as well as standardization of the training and delivery of ART. Second, the self-report outcome measures utilized are reliable and valid instruments. Third, the cohort consisted of all consecutive eligible consenting cases (*i.e.*, no “cherry picking”) which aids in generalizability of the study results. Finally, neither the founder of ART (L.R.) nor lead trainer (A.S.) performed any cases or participated in data collection or analysis.

Despite these strengths, there are several limitations. First and foremost, there was no control group to compare the results achieved with ART. This uncontrolled cohort study design is typical for an early stage evolving therapy such as ART, but can provide suggestion of effectiveness. Second, assessment of treatment efficacy of ART is based on changes in symptomatology. Thus, we cannot directly infer from our data that use of ART results in change in DSM-derived diagnoses, such as PTSD. Having said this, in the theoretical development of ART, the focus on treating images from traumatic experiences aligns closely with PTSD DSM-IV-TR Criterion B [1]; Intrusive Recollection (*recurrent and intrusive distressing recollections of the event, including images, thoughts, or perceptions*); as well as Criterion F: Functional Significance (*clinically significant distress or impairment in social, occupational, or other important areas of functioning*). Moreover, very large treatment effects were observed among participants who presented with a PCL-C score ≥54, an established optimal screening cutpoint for PTSD in public mental health settings [38]. The 2-month follow-up assessment included approximately 18% loss to follow-up which is a potential source of bias. However, the sensitivity analyses conducted showed similar initial treatment outcomes in those with and without 2-month follow-up data, and also indicated high rates of treatment response even assuming that all non-completers of treatment were non-responders. Finally, follow-up beyond 2-months and formal comparative effectiveness studies against established evidence-based psychotherapies are needed to fully assess the effectiveness of ART for treatment of PTSD.

## 5. Conclusions

From this initial assessment, ART appears to be a brief, safe, and effective treatment for symptoms of PTSD and related psychological comorbidities. Future controlled studies with ART are warranted, particularly given its short treatment duration, and in light of current heightened emphasis on health care cost constraints, as well as the very large clinical burden of treatment of PTSD being experienced from the lengthy wars in Iraq and Afghanistan.
